# Orbital Apex Metastases From Primary Colorectal Adenocarcinoma: A Rare Cause for Unilateral Vision Loss

**DOI:** 10.7759/cureus.59485

**Published:** 2024-05-01

**Authors:** Paul J Wurtz, Marcela Mazo Canola, Chandra Subedi, Olivia Fisher, Jason Lally

**Affiliations:** 1 Internal Medicine, Brooke Army Medical Center, Fort Sam Houston, USA; 2 Breast Medical Oncology, University of Texas Health San Antonio MD Anderson Cancer Center, San Antonio, USA; 3 Internal Medicine, University of Texas Health Science Center at San Antonio, San Antonio, USA; 4 Pathology, University of Texas Health Science Center at San Antonio, San Antonio, USA; 5 Radiology, University of Texas Health Science Center at San Antonio, San Antonio, USA

**Keywords:** cancer outcome disparities, uncommon metastasis, atypical colorectal cancer presentation, bony metastasis, perforated diverticulitis, young male metastatic adenocarcinoma, hispanic colorectal cancer, sigmoid adenocarcinoma, colorectal cancer, orbital metastasis

## Abstract

Colorectal cancer is one of the most common causes of cancer-related death in the United States. Although it frequently metastasizes to adjacent structures such as the liver, orbital metastases are exceedingly uncommon. Additionally, the morbidity and mortality associated with colorectal cancer appear to be shifting to a younger population, a phenomenon that is exacerbated in minority populations. We present a case of orbital metastasis from colorectal carcinoma in a young Hispanic male. This uncommon presentation of disease emphasizes the link between healthcare disparity and differential outcomes of colorectal cancer.

## Introduction

Colorectal cancer remains a major health burden in the United States, ranking as the second leading cause of cancer-related deaths in females and males in 2020 [1]. The data also indicates that Hispanic individuals face inferior outcomes when compared to the general population, necessitating targeted interventions and research to address these disparities [2-4]. Although the mortality from colorectal carcinoma is decreasing, there are increasing numbers of younger patients presenting with advanced disease, and the reason for this remains unclear [4,5]. This is particularly true in Hispanic populations, where differences in outcomes may be partially explained by advanced disease status at presentation [6]. There appears to be a trend toward a younger age of onset and differential survival, in addition to increased prevalence in Hispanic populations [7-11].   

While colorectal cancer frequently metastasizes to the liver, lungs, and peritoneum, it does not typically spread to the head and neck region [12,13]. Head and neck metastases are a rare manifestation of most primary tumors in general, with orbital metastasis, especially from colorectal cancer, occurring in exceedingly rare circumstances. This usually portends a poor prognosis and is representative of an advanced stage of disease [14,15]. While a hematogenous spread is the most likely pathway for metastasis, the reason why orbital metastasis is an uncommon phenomenon remains poorly understood.   

## Case presentation

**Figure 1:**
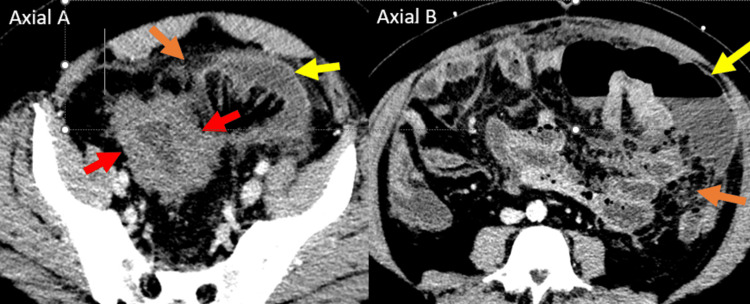
CT abdomen and pelvis with IV contrast: Axial image through the level of the sigmoid colon (A) shows wall thickening (red arrows) with surrounding fat stranding (orange arrow) and small crescentic extraluminal fluid collection (yellow arrows). A superior axial image (B) shows a larger free intraperitoneal fluid and air collection (yellow arrows) consistent with perforation.

**Figure 2:**
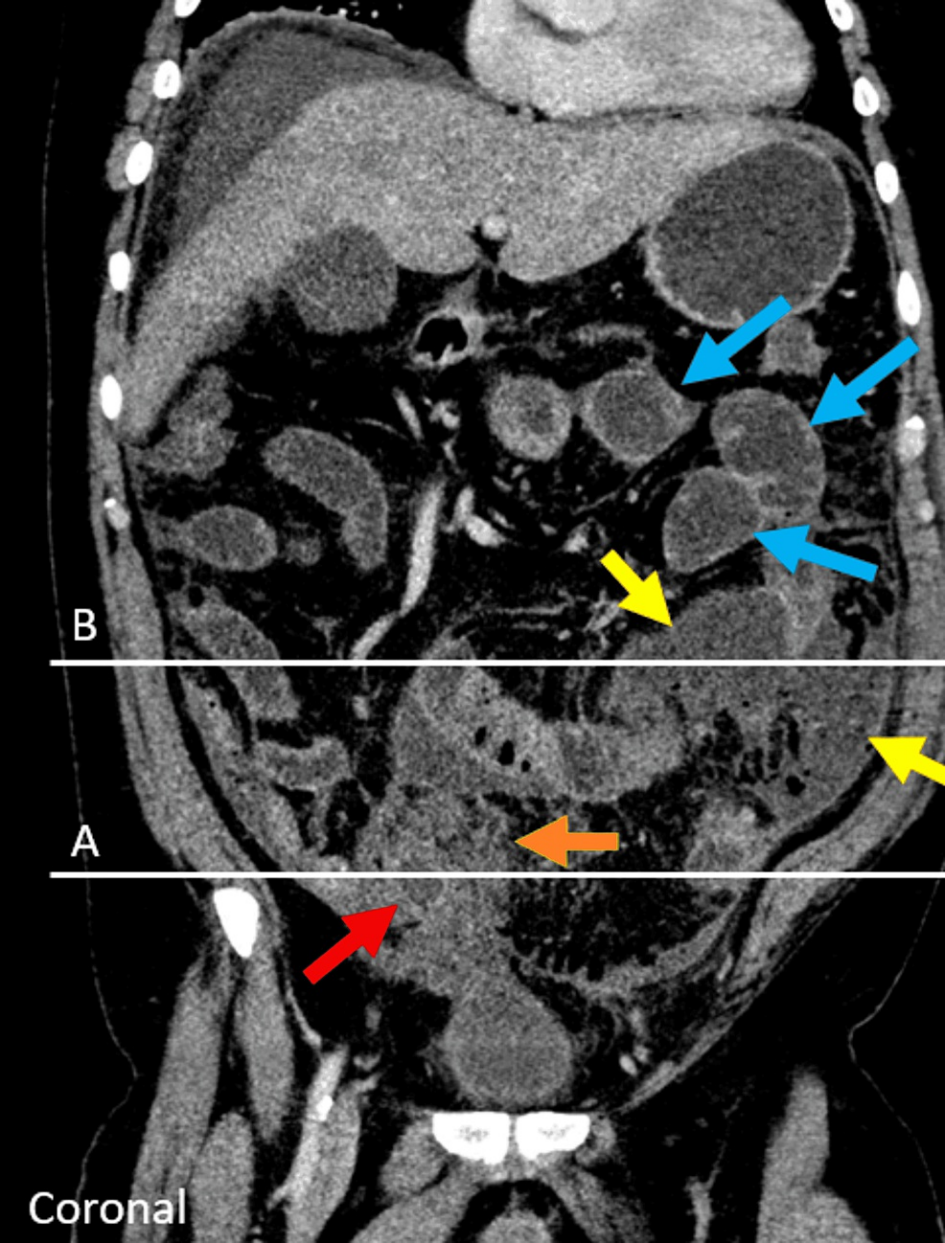
Coronal image through the abdomen shows wall thickening (red arrows) with surrounding fat stranding (orange arrow) and small crescentic extraluminal fluid collection (yellow arrows) with dilated proximal small bowel consistent with ileus (blue arrows).

A 54-year-old Hispanic male with no past medical history presented with three days of lower abdominal pain. A CT scan of the abdomen revealed evidence suggestive of perforated diverticulitis with corresponding ileus (Figures 1, 2).

The patient underwent exploratory laparotomy, with the surgeon immediately encountering purulence and adhesions to the anterior abdominal wall, adjacent to bilateral pelvis abscesses and underlying diffuse fecal peritonitis. As adhesions were freed, diffuse peritoneal implants were appreciated throughout the abdomen. The omentum was also riddled with carcinomatosis. Due to the extent of bowel involvement, the surgery was modified to accommodate a diverting-loop ostomy. Further imaging showed a small bowel obstruction likely secondary to the malignancy, and a rectosigmoid mass-like wall thickening which abutted the posterior wall of the urinary bladder. Also, a hypodense mass up to 2.8 cm in size was seen in the right hepatic lobe concerning for hepatic metastasis. Finally, ascites and diffuse omental and peritoneal thickening and nodularity, concerning for peritoneal carcinomatosis, were noted on the radiologist’s report. A biopsy done at the time of surgery demonstrated moderately differentiated mucinous adenocarcinoma cells with signet ring features. Hence, the diagnosis was confirmed: extensive metastatic colorectal adenocarcinoma complicated by peritoneal carcinomatosis and liver metastasis.   

Outpatient follow-up with medical oncology was arranged within three weeks of hospital discharge, with plans to initiate FOLFOX6 (leucovorin calcium (folinic acid), fluorouracil, and oxaliplatin). Panitumumab was to be added to the chemotherapy backbone if molecular testing revealed KRAS, NRA, BRAF, and HER2 WT status, which did not manifest. No bevacizumab was planned given his recent history of perforated diverticulitis. At his initial visit, carcinoembryonic antigen (CEA) levels were found to be 162.3 ng/mL with testing for MLH1, MSH2, MSH6, and PMS2 showing intact nuclear expression. Upon chart review, the patient was seen to have made only one follow-up appointment with oncology, with evidence of many phone cancellations prior to his visits. There were initial mentions of a lack of insurance but efforts were being made to find alternative funding. Although funding may have played a role initially, it seems through chart review and direct conversations with the patient that he did not wish to have the interventional radiology (IR) port placed for chemotherapy, as he was discussing options with his family. Unfortunately, this patient’s reluctance regarding chemotherapy lasted for only three and a half months, as he presented to the hospital again with nausea, abdominal pain, and bilious vomiting and was found to have an ileus. Oncology began following and he was started on inpatient FOLFOX chemotherapy 11 days after this second presentation.  This initial cycle was intended to target his obstructive symptoms; PET-CT was deferred to outpatient. 

**Figure 3:**
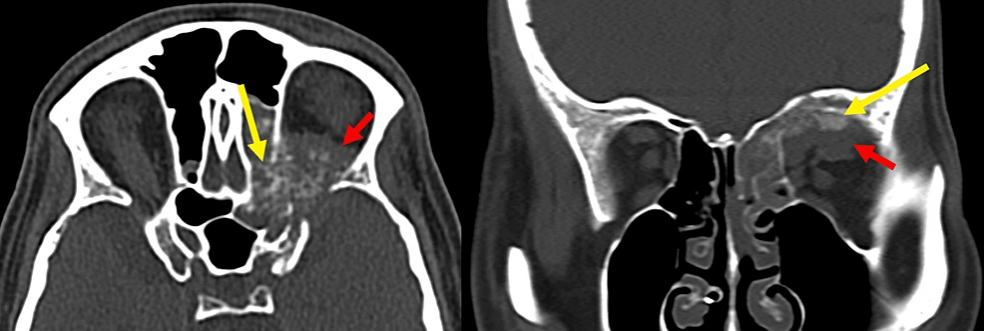
CT head without contrast: Left orbito-ethmoid expansile and lytic osseous mass with aggressive periosteal reaction (yellow arrow) and soft tissue component (red arrow). The mass involves the orbital apex and destroys the bony optic canal.

**Figure 4:**
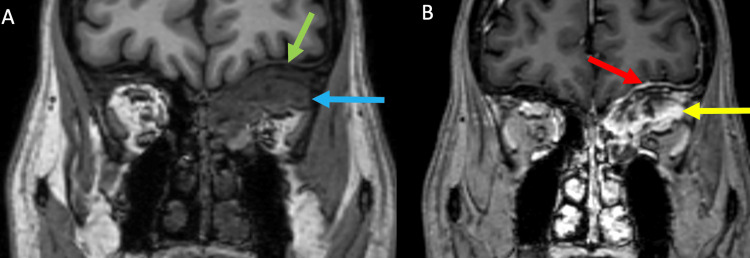
MRI brain without and with contrast: Pre-contrast T1 weighted image (A) shows replacement of the marrow (green arrow), invasion and obliteration of the superonasal extra-conal orbital fat (blue arrow), and loss of fat plane to the superior rectus muscle. After the administration of intravenous contrast (B), the mass shows heterogeneous enhancement (yellow arrow). Enhancement of the dura (red arrow) may be reactive inflammation or early tumor infiltration.

During this admission, the patient developed a progressive headache that worsened over several days. Subsequently, he was found to have unilaterally decreased visual acuity on the left side. Ophthalmological examination revealed left-sided proptosis, significant lateral and medial gaze palsy, and diplopia with medial and lateral gaze. The dilated fundus exam was limited by prominent asteroid hyalosis. A CT and MRI of the brain revealed an enhancing mass infiltrating the region of the orbital apex and exerting mass effect on the conal structures (Figures 3, 4).

**Figure 5:**
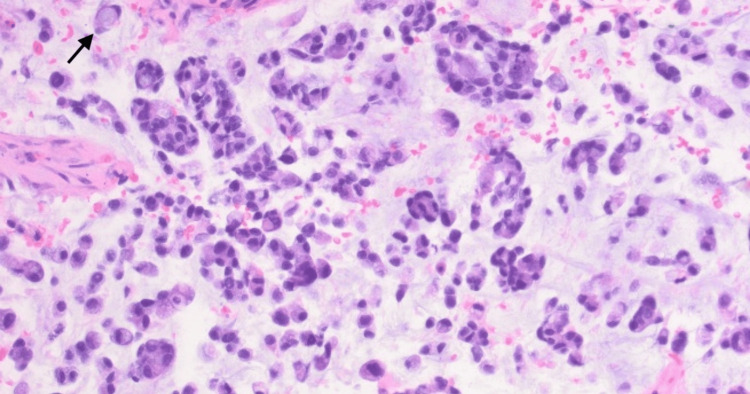
Neoplastic glands and single cells in mucin. Signet-ring cells are noted (arrow). H&E, 200x.

Due to the rarity of this location for metastasis, a biopsy was performed at the request of the specialty team. An otolaryngology specialist approached with an endoscope via the nasal cavity. They proceeded medial to the middle turbinate to the superior turbinate and identified tissue in the superior meatus which looked abnormal and hypervascular. Finally, they used biting cup forceps to take four biopsies of the tissue which revealed moderately differentiated adenocarcinoma with mucinous features consistent with metastatic colon adenocarcinoma (Figure 5).

Based on the biopsy and radiological findings, a diagnosis of metastatic sigmoid adenocarcinoma to the left orbital region was made. He was managed symptomatically while he continued with palliative FOLFOX chemotherapy. His headaches continued to worsen, and his vision rapidly deteriorated; thus, it was decided to expedite a cycle of radiation to attempt symptom relief. In the initial days following radiation, the patient mentioned symptomatic improvement. However, by this point, his disease had advanced and he was experiencing no treatment response from his chemotherapy. He underwent a total of two cycles of FOLFOX during this hospitalization but was not offered a third cycle due to a lack of clinical improvement. Due to the advanced nature of his disease, he was offered outpatient hospice services and ultimately died three weeks after undergoing radiation, six weeks from his initial visual symptoms.   

## Discussion

Despite a decline in mortality rates from colorectal carcinoma, there is a notable rise in younger patients being diagnosed with advanced stages of the disease. This is largely due to the fact that the improvement in overall colorectal cancer survival is chiefly driven by declining mortality in the geriatric population, while the younger populations are continuing to present with more advanced disease at progressively younger ages [1]. Minority populations face inferior outcomes when compared to non-Hispanic White patients in most forms of cancer including colon and rectal [16,17]. Our case aims to highlight the disparities that exist within Hispanic populations, where differences in colorectal outcomes can be partially attributed to differences in socioeconomic status and advanced state of the disease at the time of diagnosis [2,18,19]. 

This case demonstrates a highly unusual representation of a relatively common malignancy; orbital metastases are a rare but well-described phenomenon in the breast cancer literature, but there are few cases associated with colorectal cancer [20]. Most presentations consist of diplopia, blurred vision, and limited eye movement, with severe cases progressing to proptosis, vision loss, and a palpable mass [13]. The patient described in our case presented with many of the same symptoms, notably with an unrelenting headache. Radiation therapy is often used to alleviate symptoms [13], which is how our patient was treated. Through the relief from this therapy, he was able to transition to hospice more comfortably, emphasizing the importance of swift diagnosis. 

## Conclusions

This case demonstrates an unusual presentation of colorectal carcinoma in a young Hispanic male and highlights the need for further investigation into several areas. These include disparities in colorectal cancer outcomes in Hispanic populations, increasing numbers of younger patients presenting with advanced disease, and recognizing uncommon metastases in the setting of a known primary cancer. Orbital metastasis, although rare, can cause significant morbidity, affecting visual function and quality of life. This patient experienced an improvement in his symptoms following radiation, emphasizing the importance of recognizing these uncommon manifestations early. Furthermore, addressing the disparities in colorectal cancer outcomes among different populations, including Hispanic patients, is imperative to improve quality of life, overall survival, and healthcare equality. 
